# A Review of Different Stimulation Methods for Functional Reconstruction and Comparison of Respiratory Function after Cervical Spinal Cord Injury

**DOI:** 10.1155/2020/8882430

**Published:** 2020-09-17

**Authors:** Jiaqi Chang, Dongkai Shen, Yixuan Wang, Na Wang, Ya Liang

**Affiliations:** ^1^School of Automation Science and Electrical Engineering, Beihang University, Beijing 100191, China; ^2^Engineering Training Cent, Beihang University, Beijing 100191, China; ^3^Nursing Department, Juancheng County People's Hospital, Shandong 274600, China

## Abstract

**Background:**

Spinal cord injury (SCI) is a common severe trauma in clinic, hundreds of thousands of people suffer from which every year in the world. In terms of injury location, cervical spinal cord injury (CSCI) has the greatest impact. After cervical spinal cord injury, the lack of innervated muscles is not enough to provide ventilation and other activities to complete the respiratory function. In addition to the decline of respiratory capacity, respiratory complications also have a serious impact on the life of patients. The most commonly used assisted breathing and cough equipment is the ventilator, but in recent years, the functional electrical stimulation method is being used gradually and widely.

**Methods:**

About hundred related academic papers are cited for data analysis. They all have the following characteristics: (1) basic conditions of patients were reported, (2) patients had received nerve or muscle stimulation and the basic parameters, and (3) the results were evaluated based on some indicators.

**Results:**

The papers mentioned above are classified as four kinds of stimulation methods: muscle electric/magnetic stimulation, spinal dural electric stimulation, intraspinal microstimulation, and infrared light stimulation. This paper describes the stimulation principle and application experiment. Finally, this paper will compare the indexes and effects of typical stimulation methods, as well as the two auxiliary methods: training and operation.

**Conclusions:**

Although there is limited evidence for the treatment of respiratory failure by nerve or muscle stimulation after cervical spinal cord injury, the two techniques seem to be safe and effective. At the same time, light stimulation is gradually applied to clinical medicine with its strong advantages and becomes the development trend of nerve stimulation in the future.

## 1. Introduction

Spinal cord injury is a common clinical severe trauma, which will disturb the communication between the brain and the body, leading to the loss of control of other intact neuromuscular system [1]. Around the world, according to the World Health Organization, between 250000 and 500000 people suffer from spinal cord injury every year [2]. About 20% of spinal cord injuries occur between C1 and C4 levels.

After cervical spinal cord injury, the decline of pulmonary function is reflected in three areas: respiratory function, cough ability, and limitation of voice function [3], especially in respiratory function [4], which has no significance worsen in respiratory function in more than 20 years [5]. The ability to breathe requires continuous movement of the skeletal muscle [6]. The diaphragm, which is dominated by C3-C8, is the main inspiratory muscle and the external intercostal muscles, which are dominated by T1-T11, produce about 35-40% of a person's vital capacity. For inspiratory and expiratory steps [7], it is confirmed that inspiratory muscle strength has a more important effect on the respiration and cough ability of SCI patients [8, 9]; also, positive muscle contraction and the combination of abdominal wall is required for expiratory performance and adequate ventilation to maintain a higher respiratory rate and volume [10]. After spinal cord injury, the injury of neck and upper thoracic cord destroyed the function of diaphragm, intercostal muscle, pararespiratory muscle, and abdominal muscle [11], which means that the ventilation system cannot work in the best way, and muscle activity is often out of sync.

In addition to the above three areas, patients with such injuries have a high risk of respiratory complications [12]. It is reported that nearly 84% of the patients with acute hospitalized cervical spinal cord injury have respiratory complications, 20% receive tracheotomy and mechanical ventilation, and 4-5% need lifelong ventilation support [13]. Spinal cord injury can lead to respiratory muscle injury, decreased vital capacity, ineffective cough, reduced lung and chest wall compliance, and excessive respiratory oxygen consumption due to distorted respiratory system [14]. As a result, the ability of clearing respiratory secretions in patients with spinal cord injury is obviously impaired, which leads to discomfort and inconvenience and develops into atelectasis and recurrent respiratory infection [15, 16].

The most commonly used assisted breathing and cough equipment is the ventilator. The main work of ventilator-dependent spinal cord injury patients is positive pressure mechanical ventilation. In the early stage of mechanical ventilation, patients are passive breathing and provide temporary respiratory support. Due to the nature of these ventilator devices, patients cannot improve their respiratory function, increase the risk of respiratory infection [13], and also accompany with bedsore and other problems. At the same time, getting rid of the ventilator requires medication [17]. The idea of nerve stimulation to restore spinal cord function can be traced back to 1970s, but it has made remarkable progress in the 21st century [2].

Although scholars have made positive results in the research of spinal nerve stimulation, there is no summary analysis of stimulation equipment and methods. The review can be used to collect the results of different studies, so as to evaluate the therapeutic effect of various means. This review will introduce the current progress of function reconstruction after spinal cord injury and make a key overview of the reconstruction of respiratory function. At the same time, this paper compares the stimulation parameters and effects of several stimulation methods for respiratory function reconstruction and discusses the influence of auxiliary means on the treatment effect. It will also introduce the hot issues in this research field, so as to show that this field has a very high research prospect.

## 2. Methods

### 2.1. Literature Search and Selection

We searched the experimental articles of scholars who studied the functional reconstruction of neural and muscle stimulation in Google academic. The key words used for searching are spinal cord injury, nerve electrical stimulation, infrared light stimulation, respiratory function reconstruction, and cough function reconstruction. References to identified articles were also manually searched for articles that were not found in the initial search. Some articles are excluded if they are non-English, repetitive (or research participants are not independent of previous publications), meeting summaries, and editorials. The selected papers are divided into two categories: therapeutic reports and analytical reviews.

### 2.2. Data Extraction

In the systematic introduction part, stimulation method is used as a classification. In the comparison part, we choose several representative articles with complete data from each stimulation method, all of which are aiming at the reconstruction of respiratory function for comparison. The following data were extracted from the selected therapeutic report: (1) participants' condition, (2) treatment method, (3) stimulator and stimulation parameters, and (4) evaluation index.

## 3. Results

### 3.1. Muscles Stimulation

Functional electrical stimulation (FES) is a kind of technology that uses a safe level current to activate the damaged or disabled neuromuscular system for function reconstruction [18]. In a closed-loop system, the parameters of electrical stimulation are constantly modified by the computer through the feedback information of muscle strength and joint position, so as to stimulate different muscle groups at the same time, which leads to the combination of muscle contraction required for complex and complex functional activities (such as walking) [19]. The main problem related to the FES model is feedback control [18], which means more sensors are needed to measure muscle strength, muscle fatigue, joint position, angular velocity, and torso position, and all of them need microprocessor for accurate analysis. As a response to all these sensory inputs, FES system should be able to define stimulus parameters according to the feedback received, so as to provide more natural response and smoother transition.

The above theory has been confirmed by many scholars. Lane induced the recovery of ipsilateral phrenic motoneurons and phrenic muscle activity under the condition of terminal neurophysiology and found a persistent spontaneous recovery in this model [20]. Warren found that there was an endogenous plasticity mechanism after spinal cord injury to promote respiration and help restore lung ventilation. These mechanisms include activation of alternative or potential pathways, endogenous germination or synaptic formation, and possible formation of new respiratory control centers [21]. Horn pointed out that the autonomic nervous system is an attractive target for medical treatment with electronic devices due to its potential of selective control and few side effects. Courtine also summarized the progress of biological and engineering strategies in recent years to enhance neural plasticity and functional recovery in SCI animal models. Many scholars apply these theories and related medical devices to clinical experiments [22].

Diaphragmatic pacing (DP) is a minimally invasive method alternative to mechanical ventilation for the treatment of cervical spinal cord palsy patients with high cervical spinal cord injury [23]. Sieg reviewed the application of phrenic nerve stimulator in spinal hypertension and central ventricular fibrillation syndrome in 1980s [24]. DiMarco explored the feasibility of laparoscopic placement of intramuscular diaphragm electrodes for long-term ventilation support in patients with ventilator-dependent [25]. He also placed two diaphragm electrodes on each half diaphragm of five subjects and compared the advantages of intramuscular phrenic pacing and traditional phrenic nerve pacing [26]. Afterwards, he proposed several feasible DP systems, including the traditional DP system with electrodes directly placed on the phrenic nerve via thoracotomy and the minimally invasive DP system with electrodes placed in the phrenic nerve via laparoscopy [27]. Cosendai tested a RF powered pulse generator, a rechargeable battery-powered pulse generator, and the external pulse generator and determined that the implantable system can replace the external pulse generator [28]. The safety and effectiveness of this technology has also been concerned. Tedde explored the indications of inserting the DPS electrode under laparoscope and described the operation methods of five patients with quadriplegia [29]. Garara systematically reviewed the safety and efficacy of the intramuscular diaphragm stimulator in the treatment of patients with traumatic high cervical trauma who depended on a ventilator for a long time, especially security due to the insertion time of the stimulator [30]. Tarek reported that a 4-year-old child with spinal cord injury received diaphragm pacing with low amplitude, changeable pulse width gradually got rid of ventilator [31]. Dean reviewed the safety and effectiveness of DP in pediatric, described the process of ventilator disconnection and diaphragm regulation during inpatient rehabilitation, and pointed out that DP implantation is a safe and effective treatment [32]. Franco Laghi increased the expiratory flow of patients through the skin stimulation of abdominal muscles [33].

In addition to diaphragm, abdominal FES can also improve respiratory function. Based on previous experiments, McCaughey reviewed the evidence of improvement of respiratory function by abdominal FES after SCI. Moreover, FES in abdomen can still significantly improve the peak flow of cough. Electromyography is usually used to measure the recovery of cough function [34]. After abdominal FES training, there was also a significant increase in autonomic vital capacity, forced vital capacity, and maximum expiratory flow compared with baseline [35–43]. FES can also be used to reconstruct cough function. McBain stimulated abdominal muscles with surface electric stimulation, placed electrodes in the posterolateral position, and helped patients with high-level spinal cord injury clear airway secretions in combination with cough [44]. A Zupan et al. discussed the problem of improving the cough efficiency of the abdominal wall electrical stimulation through the experiment, so that the patients with high cervical spinal cord injury could get diaphragm pacing in the treatment [45]. Cheng et al. verified that the electric stimulation of thoracoabdominal muscles can improve the cough ability and lung function of patients with cervical spinal cord injury of quadriplegia [46].

On the other hand, the restoration of hand grasping function is also a research hotspot of muscle electrical stimulation. Mulcahey studied the application and functional benefits of implanted functional electrical stimulation (FES) system in patients with quadriplegia after spinal cord injury. He used five adolescents aged 16 to 18 with traumatic spinal cord injury as samples to excite the key muscles of metacarpal, lateral grasp and release through electrical stimulation, and completed the assessment of grip strength [47]. Mangold stimulated extensors and thumbs in all subjects to achieve lateral and palm grasping functions [48]. Popovic compared 21 patients with C3-C7 new spinal cord injury and found that functional electrotherapy has potential therapeutic potential, which is an effective method to restore the grasp function of patients with quadriplegia [49]. Ragnarsson summed up the existing problems and put forward the prospect that developing a fully implantable, easy to manufacture, modular FES system, which can be used for all purposes, such as upper and lower limbs, trunk, bladder, intestine, and diaphragm functions [50].

Compared with electrical stimulation, the research of magnetic stimulation is much less. Lin explored the role of functional magnetic stimulation in the conditioned reflex of the expiratory muscles of the patients with spinal cord injury [51]. Fawaz used a randomized controlled trial to compare the effects of two rehabilitation programs, one for FES and true magnetic stimulation, the other for FES and false magnetic stimulation, on the hand function recovery of patients. The results showed that patients receiving magnetic stimulation have recovered quickly [52].

### 3.2. Epidural Electrical Stimulation

It is conformed that the motor activity after spinal cord injury can be realized through the regulation of neural circuits by epidural stimulation [53]. According to Jackson [2], the idea of using electronic implants to bypass damaged neural pathways can be traced back to the 1970s [54], but significant progress has been made in this field in the 21st century [55, 56]. It is not only verified in monkeys that the brain signal can control the stimulation of the cervical region at the top of the spinal cord to restore the movement of paralyzed arms and hands [57] but also the lumbar spinal cord stimulation has achieved good results in human experiments to restore the leg to a certain degree of autonomous movement [58]. There is evidence that closed-loop stimulation can drive neural plasticity, which plays an important role in the rehabilitation of partial spinal cord injury [59].

**Figure 1:**
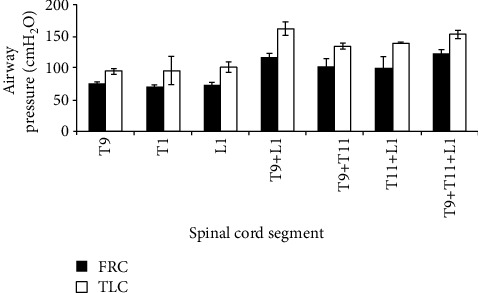
Airway pressure of different plans to activate cough.

DiMarco produced an effective coughing mechanism by stimulation of the spinal cord in the lower thoracic and upper lumbar segments. Three epidural electrodes were used in the spinal cord of T9, T11, and L1. The airway pressure was 90 cm H^2^O and 82 cm H^2^O when stimulating T9 and L1 separately, while the maximum expiratory velocity was 6.4 L/s and 5.0 L/s. When stimulation of T9 and L1 combined, airway pressure and expiratory flow rate increased to 132 cm H^2^O and 7.4 L/s, as shown in Figure 1 [60].

DiMarco completed the experiment to measure the activation ability of the lower thoracic spinal cord electrical stimulation on the expiratory muscles of the patients with quadriplegia [15]. DiMarco then proposed that the peak airflow and airway pressure generation with wire electrodes were the same as those with disk electrodes [61].

**Figure 2:**
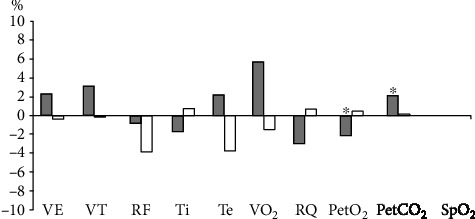
Changes of lung ventilation and gas exchange parameters during the increase of autonomic movement (gray strip) and stimulation (white strip).

Minyaeva studied the dynamic changes of pulmonary ventilation and gas exchange parameters when stimulating in T11-T12 in 10 young male subjects, as shown in Figure 2, and concluded that step motion induced by percutaneous spinal cord stimulation resulted in increased respiratory rate [62].

**Figure 3:**
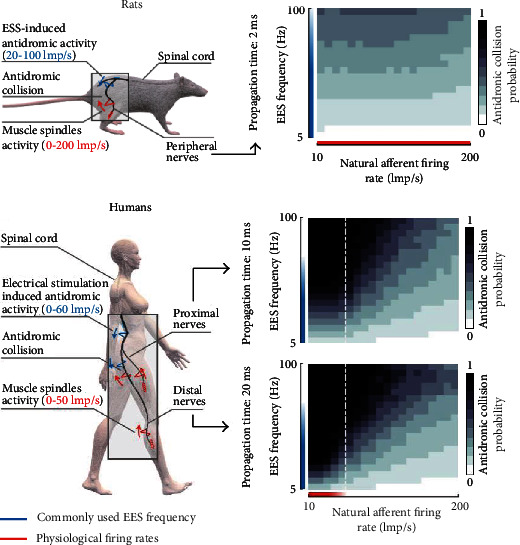
Probability of retrograde AP in sensory afferent fibers under EES.

In addition to breathing, more focus has been placed on the reconstruction of walking function. Formento explored the reasons for the differences in the effects of spinal epidural electrical stimulation in animal models of spinal cord injury and in humans. They concluded that the interspecific difference was due to the interference between EES and human ontological sensory information. Therefore, the inspiration of this study for the recovery of walking function is that sudden stimulation and spatiotemporal stimulation can reduce the elimination of proprioceptive information, so as to achieve robust control of motor neuron activity.  Figure 3 shows the comparison of the probability of reverse collision between human and mouse [63].

Harkema also pointed out that epidural spinal stimulation can regulate the spinal cord circuit to a physiological state and make the sensory input of standing and stepping motion the source of neural control. They placed a 16 electrodes array on the dura mater (L1-S1 spinal cord segment) surgically on a 23-year-old male with C7-T1 subluxated paraplegia and successfully reactivate the previously silent standby neural circuit after severe paralysis [64].

Wagner provided a series of spatial selective stimulation to the lumbosacral spinal cord by using the implanted pulse generator. After a few months, participants resumed voluntary control of previously paralyzed muscles without stimulation [65].

### 3.3. Intraspinal Microstimulation

In addition to dural stimulation, due to electrical stimulation of motor cortex that can affect the conduction of central motor tract [66], some scholars have also carried out ISMS researches. The first is the research of ISMS on muscle response, and the second is the reconstruction function of ISMS. As an interdisciplinary subject, some scholars focus on the development of ISMS equipment and the control algorithm.

**Figure 4:**
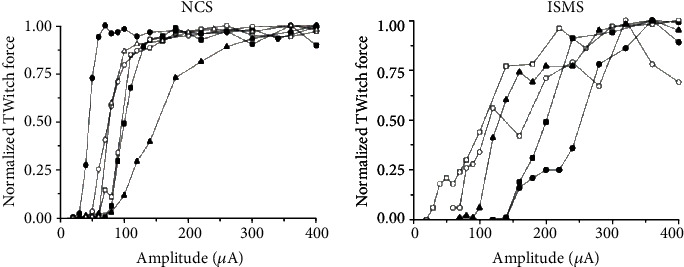
Muscle force changes after ISMS and NCS.

Moritz recorded the forelimb responses induced by cervical spinal cord stimulation that were applied in C6 to T1 sites in primates. Of the 745 stimulated areas, finger (76% of the effective area), wrist (15%), elbow (26%), and shoulder (17%) induced movement. Therefore, the stimulation usually activating multiple muscles together [67]. Mushahwar used five adult cats that fully spinal at the T12 to record the right hindlimb movements produced by muscle (*n* = 4), extraneural membrane (*n* = 2), and spinal cord (*n* = 3) stimulation. For muscle and epineurial stimulation, bipolar monophasic pulse sequences (300 microseconds, 50 pulses/second) with duration of 0.7-0.8 s were delivered through implanted electrodes at the amplitude of 0.18-4.00 mA and 16-510 *μ*A. They then concluded that motoneuron pools from in the intermediate and ventral regions can be activated by spinal cord stimulation [68]. Holinski has developed a feedback-driven isms system, which proved that ISMS can enhance the stepping function by reducing muscle fatigue and activating the spinal cord neural network to generate cooperative movement [69]. Bamford also completed an ISMS task. They activated the skeletal muscle to restore muscle activity in rats by electrically stimulating the gray matter of abdominal wall. Compared with the peripheral FES method, ISMS is easy to achieve the stable contraction level of less than 50% of the maximum spontaneous activation [70], as shown in Figure 4.

In terms of reconstruction function of ISMS, Mercier explored the ISMS in C4 spinal cord segment of adult rats after C2 hemisection in the background of respiratory movement. The stimulation mode was 250 ms (100 Hz, 100-200a) each time. The experiment was carried out successfully and induced the short-term enhancement of spontaneous inspiratory activity of 70% of the subjects, which provided the basis for the closed-loop ISMS method to maintain ventilation after severe spinal cord injury [71]. In addition, Bamford focused on the application of ISMS in the recovery of bladder function after spinal cord injury [72].

**Figure 5:**
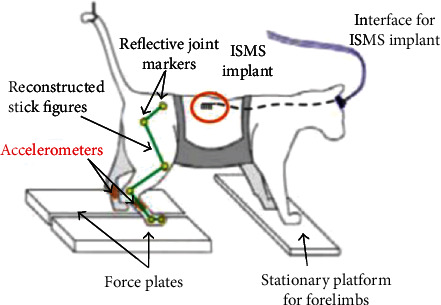
Bilateral walking experiment of activation by ISMS.

Shahboost combined the integrated circuit technology for the interface of corticospinal cord with the embedded signal processing technology based on FPGA to prove that the ISMS controlled in real time by the spike wave in the corticospinal cord can activate certain muscles of experimental rats [73] and then reported a closed-loop control method for the ISMS [74]. Troyk reported a wireless stimulator device for animal experiments. They used ISMS to activate the residual motor control neural network in the ventral horn of the spinal cord below the injury level after spinal cord injury and induced bilateral walking mode of the lower limbs. Combined with the advanced feedback algorithm, the walking distance recovered by ISMS exceeds that generated by other types of functional electrical stimulation. As shown in Figure 5, after implantation of ISMS microfilaments, the cat was placed in a safety belt and suspended on a treadmill. A microaccelerometer was placed on the legs of the cat to provide a simulated sensory feedback signal. The spinal cord of the cat was stimulated to produce a gait like pattern [75].

Because the neuromusculoskeletal system has obvious nonlinear, time-varying, large latency, and time constant as well as muscle fatigue, it is a very difficult task to control limbs accurately and stably by using ISMS. Many scholars put forward different ISMS control methods. Asadi proposed a robust control method to determine the stimulation mode, and enables the controller to compensate for the dynamic interaction between the pool of motor neurons and the electrode positions. The control method is based on the combination of sliding mode control, fuzzy logic, and neural control. A large number of experiments have been carried out on 6 rats, and the robustness, stability, and tracking accuracy of this method have been proved [76]. Roshani proposed a fuzzy logic control and used multi electrodes to study the closed-loop control of ankle motion. In order to compensate the effect of time delay, the future value of expected response was taken as the input and error signal of FLC. The results of animal experiments show that the proposed control framework can provide good tracking performance [77].

### 3.4. Infrared Light Stimulation

Near-infrared light directly irradiates the nerve tissue with infrared light, which causes the instantaneous energy accumulation in the tissue, the temperature gradient established by which can generate light and heat in the tissue, thus inducing the nerve activity [78]. Compared with traditional electrical stimulation, INS uses fiber-optic coupling laser to stimulate nerve tissue, which solves the problem of mechanical damage caused by contact electrodes in electrical stimulation; meanwhile, laser stimulation has good spatial accuracy, which can stimulate a single neuron without range effect; in addition, the stimulus signal does not affect the detected response signal because of the difference between the nature of stimulation (light signal) and response (electrical signal). It is also examined that infrared radiation increases the frequency of spontaneous synaptic events and the response size is proportional to the output of the excitation light [79].

Many scholars have done extensive research on the principle of light stimulation. Generally speaking, the interaction of light and biology includes light pressure effect, photochemistry effect, photomechanical effect, and photomagnetic effect. Richter's and Lenarz's experiments exclude the photochemical effect, because the laser used is a low-energy infrared laser [80], and the infrared photon energy is too low (<0.1 eV), and there is not enough energy to produce photochemical reaction directly. Wells et al. of Vanderbilt University exclude photomagnetic effect by using a 750 nm laser to stimulate peripheral nerves in mice. They found that the laser triggered nerve impulses may be related to the light absorption of tissues, but not to the electric field effect [80]. Then, Vanderbilt University researchers propose that photomechanical effect cannot trigger nerve impulse by observing whether the duration of the laser pulse has an impact on the stimulation threshold [81]. Wells' team used a noncontact infrared thermometer to measure the surface temperature of peripheral nerve tissue under the action of laser and found that it could cause nerve excitation when the temperature increased to 6-10°C. By analyzing the distribution of laser energy in nerve tissue, it can be concluded that about 64% of light energy is concentrated in axon, causing the temperature to rise about 3.8-6.4°C. The temperature gradient can activate the cell transmembrane ion channel and trigger neural action potential [82].

Therefore, based on the photothermal effect, some light stimulation devices have been developed and used in preliminary experiments. In the infrared stimulation of human spinal nerve root completed by Cayce and Wells of Vanderbilt University, a high-power and high-frequency clinical system light box was used to verify the safety and effectiveness of INS in human body. In 7 subjects, INS was used to stimulate two and three parts of each nerve, and electromyogram records were obtained during the whole stimulation process. At the same time, histological examination was carried out to determine the thermal damage threshold of INS. The result showed under the radiation of 0.53-1.23 J/cm^2^, 63% of the nerves appeared activation of human dorsal root, and thermal damage was found at 1.09 J/cm^2^; meanwhile, the safety ratio of 2 : 1 was determined. These findings proved the success of INS as a new way to activate human nerves in vivo and provided necessary safety data, which provided necessary impetus for the clinical application and diagnosis.

The equipment used in the experiment is the high-power high-frequency clinical system light box [83]. The high-energy laser from the optical fiber adjusted its output frequency and situation by the pulse generator and sterilized handheld bracket.

Now, it has been confirmed in many ins researches that in order to reduce the change of spot size, a fixed-point light source or a collimated beam is usually used. Fried et al. also proved that compared with the standard Gaussian beam, the collimated beam reduces the stimulation threshold and improves the reliability of the system. The advantages of the device also include that the setting of the main switch and the pulse generator increases the safety and controllability, and the external micromanipulator and the handheld bracket make it flexibly used in different positions. However, the device also has shortcomings. The handheld probe used in this study was determined as a limitation, because it is difficult to keep the distance between the probe and the tissue at 1.5 mm, which may lead to uncontrollable changes in radiation exposure. It should be more effective if a device that can accurately stabilize the probe distance was added.

**Figure 6:**
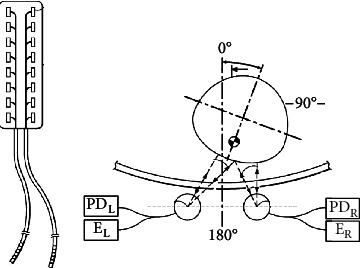
Construction and reflex measurement of spinal cord stimulation device.

As is shown in Figure 6, Wolf provided a position-sensitive NIR reflex measurement device and method for automatic regulation of spinal cord stimulation. The system consists of an electrode assembly and an integrated optical fiber sensor for sensing spinal cord position. The integrated optical fiber sensor includes a set of optical elements for emitting light from a set of infrared transmitters and collecting the reflected light into a set of infrared photodetectors to determine a set of measured light intensity. With the change of spinal cord position, the incident angle of light from infrared transmitter and the measured light intensity also change. Then, the device adjusts the pulse characteristics of the electrode in real time [84].

In addition to the experiments in human body, some light stimulation devices are also used in animals such as rats. Entwisle et al. have detected the membrane and synaptic responses of solitary tract neurons recorded in acute sections of rats. They used a 1890 nm compact waveguide laser to stimulate neurons and send light through a single-mode fiber to a small target the size of a single cell and found that the response was proportional to the laser output [79]. A thulium-doped glass waveguide laser is used in the experiment. The laser is customized by internal facilities. The output light of 1.89 *μ*m is coupled to the single-mode fiber with 14 *μ*m mode field diameter, and the nominal power output of 7.8 mw is measured at the fiber end. The fiber was placed in the micromanipulator 45 meters away from the water plane and placed about 100 *μ*m away from the target cell. The external digital trigger was used to control the power output and pulse length of the laser. In a complete one-second pulse, the pulse energy calculated on the cell surface is 261 J/cm^2^, and the spot size calculated after the light divergence is 42.8 *μ*m × 26.7 *μ*m, which shows that the device with this structure can be used for a specific single cell. This device can be improved and applied in clinic.

## 4. Discussion

### 4.1. Experimental Comparison of Different Stimulation Methods

I selected 5 cases about the recovery of diaphragm function by stimulation, involving 66 people in total, most of them were in C4-C6 spinal cord segment, and a few of them had T4-T6 injury. The age of these patients ranged from 16 to 67, most of them were men, and some of them had smoking history. The causes include gunshot wounds, car accidents, and falls. The participants are shown in Table 1.

The first, third, fourth, and fifth experiments were electric stimulation; the second was magnetic stimulation; the first and second were spinal nerve stimulation; the third, fourth, and fifth were muscle stimulation. Different stimulation methods and locations will result in different stimulation parameters and results. The duration of stimulation in spinal nerve electrical stimulation is only 5-10 minutes, while the rest is no less than 20 minutes. The stimulator of spinal nerve is placed between T9 and L1, while others are placed in muscle. The maximum stimulation frequency of these experiments is no more than 50 Hz. The pulse width of spinal cord stimulation experiment is lower than that of muscle stimulation. Authors in the first study use voltage to measure the stimulus intensity, while the fifth study used the current. In the selection of stimulator, electrodes were used in spinal cord electrical stimulation, while circular magnetic coil (outer diameter 20 cm) was used in spinal cord magnetic stimulation. Two pairs of surface electrodes were selected for muscle electrical stimulation. In the fourth study, the anterior electrode was placed in the rectus abdominis muscle under costal margin and above pubic symphysis, and the ventrolateral electrode was at the intersection of the inferior margin of costal margin and axillary midline, above the anterior superior iliac spine.

Generally speaking, there are many indexes to evaluate respiratory and cough function. The indexes used in the five cited experiments can be classified as follows. (1) Spirometry: peak expiratory flow (PEF) is the maximum rate of air exhaled from the lungs as hard and fast as possible when measured from the total vital capacity, while flow of peak cough (CPF) is the maximum rate of air exhaled from the lungs when coughing. The maximum expiratory pressure (MEP) refers to the maximum pressure generated in the mouth to resist the blocked airway when exhaling from the total lung capacity. Pressure of gastric (PGA) and pressure of esophageal (PES) can be used to measure the pressure of pleura when expiratory muscles contract. These indexes can indicate the strength of expiratory muscles.VC, FVC, and FEV1 can be used to evaluate the strength of vital capacity. These evaluation indexes are characterized by the need for active control of breathing, as well as subjects' prediction and motivation, noninvasive. Spirometry has been used in all five studies. (2) Plethysmography: parameters mentioned are respiratory rate (F), tidal volume (VT), and minute ventilation volume (VE). Only the fourth study clearly indicated that tidal volume testing was carried out. (3) Electromyography: electromyography reflects muscle activity, which is characterized by the fact that long-term electrode placement can be used for repeated measurement. Electrodes are often placed on the skin surface, which reduces the accuracy of recording. EMG electrodes were applied to the left lateral oblique muscle in the fourth study. (4) Diaphragm compound motor action potential (CMAP): the electrode is placed on the skin, and the nerve function is indirectly measured by evaluating the nerve integrity. None of the five studies used this evaluation method.

By comparing the five experiments, it can be found that the PEF-TLC of the first group is 5.8-8.8 L/s, while the magnetic stimulation is only 4.3 L/s. When referring to MEP-TLC, the first group is 120-150 cm H_2_O, while the average of the second group is 55.3 cm H_2_O. When referring to PEF-FRC and MEP-FRC, it can also be seen that although the magnetic stimulation treatment has made progress compared with nonstimulation, it is far less than that of electric stimulation. Direct stimulation of spinal cord was better than muscle stimulation. The PEF-TLC of the first study (single stimulus point: 5.8-8.6 L/s; double stimulation points: 7.8-8.8 L/s) was greater than that of the fourth study (increased from 5.1 ± 0.6 L/s to 6.1 ± 0.5 L/s) and the fifth study (4.24 L/s), which were muscle stimulation studies. The results of the last study and the second study were similar in FVC (2.5 ± 0.1 L in the second study and 2.51 L in the fifth study), FEV1 (2.0 ± 0.1 L in the second study and 2.3 L/s in the fifth study), and PEF-TLC (4.3 ± 0.5 L/s in the second study and 4.24 L/s in the fifth study), which indicated that the effect of magnetic stimulation of spinal cord was less than that of electrical stimulation of spinal cord, but similar to that of electrical stimulation of muscle. In addition, in terms of complications, 5 subjects in the first study had mild edema in the test site, while in the fifth study, one case (7.7%) in the treatment group had pulmonary complication. It is difficult to see the effect of different stimulation situations on complications.

### 4.2. Auxiliary Treatments of Functional Reconstruction

Many scholars added training as auxiliary treatment in the experiment of functional reconstruction of spinal cord injury. Boswell explored the feasibility of respiratory muscle training (RMT) as an improvement of lung function in patients with cervical spinal cord injury [85]. Tamplin explored the effect of RMT on pulmonary function in patients with quadriplegia [86]. Paleville combined 80 exercises with electrical stimulation of L1-S1 spinal cord. It is concluded that the combination of task-specific training and epidural stimulation may improve the vascular fitness and body composition of patients with cervical or upper thoracic spinal cord injury [87]. Field evaluated the effects of body weight support (BWS), FES, and treadmill training on ground walking speed, treadmill walking speed, and distance, and concluded that in the training process, all indexes were significantly improved [88]. Gee proved the effect of RMT on strengthening respiratory muscle strength, reducing exercise lung capacity, and increasing exercise ability through training disabled athletes with cervical spinal cord injury [89]. By analyzing the vital capacity of patients with SCI, Tiftik compared the effect of combination of exercise training and simple rehabilitation only program on the lung function of SCI patients and emphasized the importance of exercise training [90]. Stenson evaluated the effect of expiratory muscle training on pulmonary function in patients with spinal cord injury and got the conclusion that resistance training group has a good pulmonary function effect [91]. Liaw did similar experiments and obtained that RMT can improve respiratory function, respiratory resistance, and dyspnea of patients with cervical spinal cord injury [92]. Roy pointed out that the plasticity of repetitive training can expand the rehabilitation methods [93].

In addition to the auxiliary effect of training, the improvement of surgical methods can also greatly improve the cure rate. Nandra extended phrenic pacing treatment to paraplegia patients with high cervical spinal cord injury between C3 and C5. Four patients with high cervical spinal cord injury were selected as the study object. Each patient had phrenic nerve deficiency and received intercostal phrenic nerve transplantation and phrenic nerve pacemaker implantation. Then, it is found that patient can breathe well with diaphragm [94]. Yang suspended the posterior rib on the lower angle of the scapula with titanium cable and suspended the muscle and myofascial tissue in the area under the scapula. This method partially restored thoracic respiration in patients with high CSCI, thus improving respiratory, cough, and expectoration functions [13]. Tedde implanted laparoscopic diaphragm pacemaker in 5 cases of high cervical traumatic spinal cord injury [95].

## 5. Conclusion

According to the results of our study, we may conclude that, although the evidence is presently limited, spinal nerve stimulation and muscle stimulation are very effective in the recovery of respiratory function impairment caused by cervical spinal cord injury. Both magnetic stimulation and electrical stimulation can improve the airflow rate and airway pressure trend to a normal state, but the effect of magnetic stimulation is not as good as electrical stimulation, while the effect of muscle stimulation is not as good as spinal nerve stimulation. In addition, the treatment device has its own characteristics according to its components and function content.

At the same time, the research means and research tools of functional light stimulation are also used for reference. Light stimulation is gradually applied to clinical medicine with its strong advantages. In the experiment of functional reconstruction of spinal cord injury, training and improved surgical methods can be used as adjuvant treatment. It can be predicted that in the future, the research on spinal cord electrical stimulation and nerve light stimulation after cervical spinal cord injury is promising and has a positive effect on the treatment of respiratory injury caused by cervical spinal cord injury.

## Figures and Tables

**Table 1:** Characteristics of participants of included studies.

Num	Author	Country or region	Aetiology(SCI level)	Number of participants	Mean of age	Male (%)	Injury time
1	Anthony F. DiMarco [15]	USA	SCI(C3-C6)	9	41	89	13.11 years
2	Vernon W. Lin [51]	USA	C4-C7 T5	8	51.25	100	17.75 years
3	A Zupan [45],	Slovenia	C4-C7	13	26.9	84.62	7 months
4	Franco Laghi [33]	USA	C5-C7 T4-T6	10	47.3	Unknown	10.66 years
5	Pao-Tsai Cheng [46]	Taiwan	C4-C7	26	34.7	84.62	In 3 months
